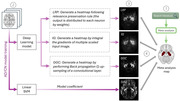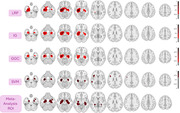# Quantifying Deep Learning Interpretability in Alzheimer's Disease Research: Validating Deep Learning Heatmaps through Meta‐Analysis

**DOI:** 10.1002/alz.089248

**Published:** 2025-01-09

**Authors:** Di Wang, Nicolas Honnorat, Anoop Benet Nirmala, Peter T Fox, Sudha Seshadri, Mohamad Habes

**Affiliations:** ^1^ UT Health San Antonio, San Antonio, TX USA; ^2^ Glenn Biggs Institute for Alzheimer’s & Neurodegenerative Diseases, University of Texas Health Sciences Center at San Antonio, San Antonio, TX USA; ^3^ University of Texas Health San Antonio, San Antonio, TX USA

## Abstract

**Background:**

Deep learning has shown promising results regarding Alzheimer’s disease (AD) studies. In the meantime, heatmap methods have emerged as a popular tool to visualize deep learning models, enhancing the transparency and explainability of deep learning. However, when no ground truth is available, the heatmap methods are particularly difficult to trust. In the neuroimaging field, meta‐analysis is often conducted to reduce heterogeneity and reveal the true positive. Thus, in this work, we aim at validating heatmap methods by quantifying the overlap of a ground truth map provided by a large meta‐analysis and heatmaps derived by three methods, the Layer‐wise Relevance Propagation (LRP) method, the Integrated Gradients (IG) method, and the Guided grad‐CAM (GGC) method from convolutional neural network achieving state‐of‐the‐art classification performance for AD classification on a sample of MRI scans from ADNI.

**Method:**

502 ADNI participants were included in this study with 250 AD participants and 252 healthy controls. Those brain scans were trained by five different 3D CNN architectures. Three CNN heatmap methods were applied on trained models to produce a heatmap indicating which voxels were the most important when classifying AD and control participants. The structural MRI meta‐analysis map used as the ground truth summarized 77 neuroimaging studies reporting 773 locations in the brain affected by AD.

**Result:**

The best CNN 5‐fold cross validated accuracy reached 87.25% for classifying AD and CN. The best Dice overlap measured for LRP, IG, and GGC, using this model was 0.502 for LRP heatmap, 0.55 for the IG heatmap, and 0.54 for the GGC heatmap. SVM activation patterns achieved a dice of 0.363.

**Conclusion:**

We found that the three heatmap methods capture brain regions that overlap well with the meta‐analysis map, and we observed the best overlap for the IG method. All three heatmap methods outperformed linear SVM models suggesting that the deep feature learnt by the most recent deep neural networks can produce more meaningful representations than linear and shallow models.